# Molecular and biological characterization of hepatitis B virus subgenotype F1b clusters: Unraveling its role in hepatocarcinogenesis

**DOI:** 10.3389/fmicb.2022.946703

**Published:** 2022-07-27

**Authors:** María Mercedes Elizalde, Laura Mojsiejczuk, Micaela Speroni, Belén Bouzas, Luciana Tadey, Lilia Mammana, Rodolfo Héctor Campos, Diego Martín Flichman

**Affiliations:** ^1^Instituto de Investigaciones Biomédicas en Retrovirus y Sida (INBIRS), CONICET, Universidad de Buenos Aires, Buenos Aires, Argentina; ^2^Consejo Nacional de Investigaciones Científicas y Técnicas (CONICET), Buenos Aires, Argentina; ^3^Departamento de Microbiología, Inmunología, Biotecnología y Genética, Cátedra de Virología, Facultad de Farmacia y Bioquímica, Universidad de Buenos Aires, Buenos Aires, Argentina; ^4^Unidad de Virología, Hospital de Infecciosas “Francisco J. Muñiz”, Buenos Aires, Argentina

**Keywords:** hepatitis B virus, subgenotype F1b, clusters, characterization, hepatocarcinogenesis

## Abstract

Hepatitis B virus (HBV) subgenotype F1b infection has been associated with the early occurrence of hepatocellular carcinoma in chronically infected patients from Alaska and Peru. In Argentina, however, despite the high prevalence of subgenotype F1b infection, this relationship has not been described. To unravel the observed differences in the progression of the infection, an in-depth molecular and biological characterization of the subgenotype F1b was performed. Phylogenetic analysis of subgenotype F1b full-length genomes revealed the existence of two highly supported clusters. One of the clusters, designated as gtF1b Basal included sequences mostly from Alaska, Peru and Chile, while the other, called gtF1b Cosmopolitan, contained samples mainly from Argentina and Chile. The clusters were characterized by a differential signature pattern of eight nucleotides distributed throughout the genome. *In vitro* characterization of representative clones from each cluster revealed major differences in viral RNA levels, virion secretion, antigen expression levels, as well as in the localization of the antigens. Interestingly, a differential regulation in the expression of genes associated with tumorigenesis was also identified. In conclusion, this study provides new insights into the molecular and biological characteristics of the subgenotype F1b clusters and contributes to unravel the different clinical outcomes of subgenotype F1b chronic infections.

## Introduction

The hepatitis B virus (HBV) infection can lead to a wide spectrum of clinical outcomes ranging from asymptomatic hepatitis to cirrhotic liver disease and hepatocellular carcinoma (HCC). Chronic HBV infection causes nearly 40% of all HCC cases worldwide (Llovet et al., 2016; Stanaway et al., 2016).

The long-term progression of chronic infection hinges on a finely balanced and complex interaction between the virus and the host immune system. The most important viral factors implicated in this interaction include HBeAg status, viral genetic variation and genotypes (McMahon, 2009; Araujo et al., 2020).

Hepatitis B virus is currently grouped into nine genotypes (designated A to I) and one putative genotype (J), based on a full genome diversity of more than 7.5%. In addition, phylogenetic analyses have shown that most genotypes have such a diversity to be categorized into subgenotypes differing by at least 4% of their genome sequence (Kramvis, 2014; Sunbul, 2014; Velkov et al., 2018; McNaughton et al., 2020).

There is growing evidence that genotypes and subgenotypes (gt) play a role influencing disease progression, including risk of developing chronic infection, HBeAg seroconversion rate, transmission mode, severity of liver disease, and antiviral treatment (Datta et al., 2018; Khatun et al., 2018). In addition, it has also been described that HBV genotypes and subgenotypes also possess differential biological characteristics *in vitro* (Sozzi et al., 2016; Elizalde et al., 2021).

The HBV genotypes show different geographical distribution in populations around the globe (Tong and Revill, 2016; Araujo et al., 2020). In particular, genotype F is autochthonous from the Americas, it has been found in native populations from Alaska, Central and South America, and it is the most prevalent in admixed populations with Native American ancestry (Rodrigo et al., 2016; Mojsiejczuk et al., 2020).

Several studies have associated subgenotype F1b infection, particularly in Alaska and Peru, with an early and rapid progression of liver disease, and evolution to HCC (Livingston et al., 2007; Machado et al., 2010; Mizushima et al., 2010; Pineau et al., 2018; Pujol et al., 2020; McMahon et al., 2021). However, despite the high prevalence of subgenotype F1b in Argentina (Ledesma et al., 2015; Rodrigo et al., 2016), HBV infection is not frequently related with the development of HCC (Fassio et al., 2009; Piñero et al., 2018), and an association of subgenotype F1b infection with a more severe progression of liver disease has not been reported.

Recently, we tested the hypothesis that the genotype F long-term evolution took place in co-divergence with prehistoric humans in Central and South America for thousands of years. Phylogeographic analysis indicated that the most probable location for the subgenotype F1b ancestor was Peru and from there spread throughout South America and to some specific populations of North and Central America. The analysis also revealed that basal divergent events within the subgenotype F1b occurred more than 2000 years ago, giving rise to highly supported groups, each formed by sequences from the same geographic location (Mojsiejczuk et al., 2020).

To unravel the observed differences in the progression of the infection, an in-depth molecular characterization of the subgenotype F1b was carried out, as well as the *in vitro* characterization of the subgenotype clusters, comparing their replicative capacity and antigen expression levels. Likewise, the role of the subgenotype F1b clusters in the modulation of cellular pathways associated with carcinogenesis was addressed.

## Materials and methods

### Phylogenetic analysis

Relationships between the HBV sequences were inferred using the Maximum likelihood method. Subgenotype F1b full-length sequences derived from HBV chronically infected patients were retrieved from GenBank (*n* = 99), and subgenotype F1a sequences were used as outgroup (*n* = 2). Dataset was built and edited in BioEdit v7.1.3.0 and aligned with Muscle v3.7 with default parameters. The best-fit evolutionary model (GTR + F + R4) was determined using Model Finder according to the Bayesian information criterion. Phylogenetic reconstruction was performed with the IQ-TREE v1.6.10 in the CIPRES Science Gateway server (Miller et al., 2010). Group support was evaluated by a standard bootstrap procedure (1,000 pseudo-replicates). Genetic pairwise distances matrix was performed by using DNADIST from BioEdit (7.05.8). Sequence numbering was according to the subgenotype F1b reference sequence HM585194 as described by McNaughton et al., 2020.

### Viral constructs

Vector pUC19 containing full-length genomes of HBV gtF1b Cosmopolitan and gtF1b Basal clusters were constructed for this study. The samples closest phylogenetically to the consensus of each cluster were selected for cloning. Briefly, HBV DNA was extracted from the HBeAg positive serum samples and full-length genomes were amplified, adapting the method described by Günther (Günther et al., 1995). For directional cloning of the PCR products P1 sense primer was modified to contain the *NdeI/BspQI* sites: 5′-CCGGACATATGATGCTCTTCTTTTTCACCTCTGCCTAATCATC-3′, and P2 antisense primer was modified to contain the *SacI/BspQI* sites: 5′-CCGGAGAGCTCATGCTCTTCAAAAAGTTGCATGGTGCTGGTG-3′. PCRs were performed using the Expand high-fidelity PCR system (Roche, Mannheim, Germany) in accordance with the manufacturer’s instructions.

The amplified HBV DNAs were digested with *Nde*I and *Sac*I restriction enzymes (New England Biolabs, Beverly, MA, United States) and separated by gel electrophoresis. The 3.2 kb fragments were recovered by gel purification with the PureLink Quick Gel Extraction Kit (Invitrogen, Carlsbad, CA, United States) and inserted into the *Nde*I/*Sac*I sites of pUC19 vector to generate gtF1b Cosmopolitan and gtF1b Basal plasmids. All constructs were verified by DNA sequencing. In order to mimic viral diversity, a mix of 15 clones of each cluster were used in all experiments. Complete genome sequences were deposited in GenBank under accession numbers: OK106257 (gtF1b Cosmopolitan) and OL907123 (gtF1b Basal).

### Cell culture and transfection

Human hepatoma cell lines HuH-7 (JCRB Cell Bank #0403) and HepG2 (ATCC HB-8065) were grown in Dulbecco’s modified Eagle’s medium (DMEM; Sigma, CA, United States) supplemented with 10% fetal bovine serum (Sigma, CA, United States), 1 mM nonessential amino acids (GIBCO, Carlsbad, CA, United States), 2 mM L-glutamine (GIBCO, Carlsbad, CA, United States), 0.15% sodium bicarbonate, 100 UI/mL penicillin and 100 μg/ml streptomycin. Cells were maintained at 37°C in a humidified atmosphere containing 5% CO_2_.

Full-length linear HBV DNAs (nt 1820-1820) with sticky ends were used for transfections. Briefly, linear HBV monomers were excised from the pUC19 plasmids by restriction with *Bsp*QI (New England Biolabs, Beverly, MA, United States) at 50°C. The 3.2 kb fragments were gel purified and the DNA was quantified by spectrometry.

Cells were seeded to semi confluence in 6 or 24 well plates and transfected with 1 μg of full-length HBV DNAs for 6 well plates and 0.25 μg for the 24 well plates. Transient transfections were carried out using X-tremeGene™ 9 transfection reagent (Roche, Mannheim, Germany), according to the manufacturer’s recommendations. After 6 h, the medium was replaced, and the cultures were incubated for 72 h.

After transfection, HBV lineal genomes with sticky ends are circularized in the cell nucleus using host cell enzymes, which can effectively allow the formation of cccDNA and promote HBV life cycle in the host cell. This experimental approach, despite its limitations, has been widely endorsed for the biological characterization of HBV (Günther et al., 1995; Allweiss and Dandri, 2016).

In all experiments, 0.05 μg of the luciferase reporter vector pGL4.13 [luc2/SV40] (Promega, Madison, WI, United States) was co-transfected with the linear full-length HBV genomes as transfection efficiency control. Results were expressed per relative light units.

### Analysis of covalently closed circular DNA

HBV cccDNA detection was performed by Southern blot and quantitative Real-Time PCR (qPCR). For Southern blot detection, a modified Hirt extraction procedure was used. Briefly, 72 h post-transfection, cells were treated with 1.5 ml lysis buffer (10 mM Tris–HCl (pH 7.5), 10 mM EDTA, 1% SDS) for 30 min, followed by addition of 0.4 ml of 5 M NaCl and incubation at 4°C overnight. The lysate was then clarified by centrifugation at 14,500 g for 30 min at 4°C and extracted with phenol and phenol-chloroform. DNA was precipitated with two volumes of ethanol and dissolved in TE buffer. DNA was separated on a 1.2% agarose gel and blotted onto a nylon positive membrane (Roche, Mannheim, Germany). The DNA was immobilized by an ultraviolet crosslinker and hybridized with a subgenomic digoxigenin (DIG)-labeled probe (Roche, Mannheim, Germany). The hybridization signals were detected using an enzyme-linked immunoassay (DIG Luminescent Detection Kit; Roche, Mannheim, Germany).

For qPCR quantification, 72 h post-transfection, cells were treated with lysis buffer containing 50 mM Tris–HCl (pH 8), 10 mM EDTA, 100 mM NaCl, 0.5% SDS, and 0.5 mg/ml proteinase K (Invitrogen, United States), and incubated at 56°C for 2 h. Nucleic acids were purified by phenol-chloroform extraction and ethanol precipitation. To prevent non-specific amplification of non-covalently closed circular forms of HBV-DNA, isolated intracellular total DNA was treated with 500 U/ml of T5 exonuclease (New England Biolabs, Beverly, MA, United States) at 37°C for 1 h to remove non-supercoiled dsDNA, followed by heat-inactivation at 95°C for 5 min and fourfold dilution with distilled water. Selective cccDNA primers: sense 5′-GTCTGTTCCTTCTCATCTGC-3′ (nt 1,551 to 1,570) and antisense 5′-AGGCACAGCTTG GTGGCTTG-3′ (nt 1887 to 1868) were used for the qPCR. Mitochondrial DNA was analyzed as an internal reference to normalized cccDNA levels. The primers for detection of mitochondrial DNA were: sense 5′-CCCCACAAACCCCATTACTAAACCCA-3′ and antisense 5′-TTTCATCATGCGGAGATGTTGGATGG-3′. Serial dilutions of an HBV replication-competent plasmid (pCH-9/3091) were used as quantification standards. HBV DNA extracted from serum samples of HBeAg positive patients with a viral load of 8.2 Log10 IU/ml was used as a control to evaluate the efficacy of DNase treatment and the specificity of the cccDNA amplification.

### Analysis of HBV RNA

HBV RNA was analyzed by Northern blot. Total cellular RNA was extracted with TRIzol reagent (Invitrogen, Carlsbad, CA, United States), according to the manufacturer’s recommendations. RNA concentration was determined by spectrometry, and 15 μg were denatured in loading buffer at 65°C for 10 min and separated in 1.5% agarose gel with morpholinepropanesulfonic acid (MOPS) and formaldehyde. After transfer onto a nylon positive membrane (Roche, Mannheim, Germany), the blots were hybridized with a digoxigenin (DIG)-labeled HBV-specific probe. RNA signal was detected by DIG Luminescent Detection Kit (Roche, Mannheim, Germany).

### Analysis of intracellular HBV DNA

HBV replicative intermediates were analyzed by Southern blot. Briefly, 72 h post-transfection, cells were treated with lysis buffer (50 mM Tris/HCl pH 7.5; 100 mM NaCl; 1 mM EDTA; 0.5% NP40) and centrifuged 1 min at 12,000 g to remove the nuclei. The supernatants were collected and treated with 0.5 mg/ml proteinase K (Invitrogen, Carlsbad, CA, United States) at 56°C for 2 h. Nucleic acids were purified by phenol-chloroform extraction and ethanol precipitation. Southern blotting was performed as described above.

### Analysis of extracellular HBV DNA

Viral progeny was quantified by qPCR. Seventy-two hours’ post-transfection, cell culture supernatants were harvested and clarified by centrifugation at 3,000 g for 10 min. Supernatants were treated with lysis buffer (50 mM Tris–HCl pH 7.5; 1 mM EDTA; 1% SDS; 0.5 mg/ml proteinase K (Invitrogen, United States)) and incubated at 56°C for 2 h. Nucleic acids were purified by phenol-chloroform extraction and ethanol precipitation. The following primers that specifically amplify relaxed-circular HBV DNA were used for the amplification: sense 5′-ATGGAGACCACCGTGAACGC-3′ (nt 1,608 to 1,627) and antisense 5′-AGGCACAGCTTG GTGGCTTG-3′ (nt 1887 to 1868).

To rule out a possible amplification of the input DNA, serial dilutions of the DNA used for transfection were analyzed. A specificity of 5 logs was observed. Serial dilutions of an HBV replication-competent plasmid (pCH-9/3091) were used as quantification standards.

### Antigen quantification

HBsAg concentration in cell lysates and culture supernatants was measured by electrochemiluminescence immunoassay (ECLIA) using the Elecsys HBsAg II quant II on a Cobas e801 instrument (Roche, Mannheim, Germany). Results were expressed in IU/ml. HBeAg concentration in cell lysates and supernatants was measured using the Elecsys HBeAg (Roche, Mannheim, Germany), in accordance with the manufacturer’s instructions. Results were expressed in Sample/Cut off value (S/CO).

The linearity dynamic range of the assay was validated making serial dilutions of serum samples with known HBeAg levels. Furthermore, the possible interference of the intracellular cell lysate in the specificity of the assay was evaluated by challenging serum samples with known HBeAg or qHBsAg levels diluted with non-transfected cell lysates.

### Western blot analysis

For intracellular HBV surface proteins, cells were lysed with RIPA buffer in the presence of protease inhibitor cocktail (Sigma, United States). For extracellular proteins, supernatants were used directly. Samples were mixed in Laemmli buffer, boiled, loaded on 12% SDS-polyacrylamide gels, and transferred to PVDF membranes (Hybond, GE Healthcare, United Kingdom), according to standard protocols. HBV surface proteins were detected using anti-SHBs (HB01) monoclonal antibody (kindly provided by Prof. Aurelia Zvirbliene, Lithuania). Peroxidase-conjugated anti-mouse secondary antibodies were purchased from Santa Cruz Biotechnology (United States). Protein specific bands were visualized using an enhanced chemiluminescence (ECL) system (GE Healthcare, United Kingdom). Quantification was performed using ImageJ software (Wayne Rasband, NIH, United States). β-actin detection was used as intracellular protein loading control.

### HBV promoter activity

To evaluate the activity of the viral promoters, Luciferase reporter vectors were built. Briefly, HBV DNA fragments covering the Core Promoter (CP, nt 1,408–1,885), Surface Promoter 1 (SPI, nt 2,458–2,858) or Surface Promoter 2 (SPII, nt 2,920–3,163) regions were amplified by PCR from gtF1b Cosmopolitan and gtF1b Basal clones with primers including *SacI* and *NheI* restriction sites (Supplementary Table 1). PCRs were performed using the Expand high-fidelity PCR system (Roche, Mannheim, Germany) in accordance with the manufacturer’s instructions. The amplified HBV DNAs were digested with *SacI* and *NheI* restriction enzymes (New England Biolabs, Beverly, MA, United States) and inserted into the *Sac*I/*Nhe*I sites of pGL4.10[luc2] basic vector (Promega, Madison, WI, United States). All constructs were verified by DNA sequencing.

Mutation of the TATA box-like sequences of the Precore and pgRNA promoters were made by a two-step, PCR-based directed mutagenesis method, as previously described (Yu and Mertz, 1996). Briefly, two outer primers carrying wild-type Core promoter sequences (CP_Fw and CP_Rv) and two pairs of complementary inner primers, carrying Precore (PcMut_Fw and PcMut_Rv) or pgRNA (PgMut_Fw and PgMut_Rv) mutated sequences were used (Supplementary Table 1). Initially, two separate PCRs using one internal and one external primer were performed, resulting in a DNA product containing the desired mutation in either the 3′ or the 5′ end. After purification, the amplification products were used as a template for the second PCRs with the external Core promoter primers. The resulting amplification products, carrying the desired mutations, were subcloned into the *Sac*I/*Nhe*I sites of pGL4.10[luc2] basic vector (Promega, Madison, WI, United States). All promoter mutations were confirmed by DNA sequence analysis.

Cells were transfected with the different constructs, and 72 h post-transfection the light emission produced by the luciferase activity was determined with the Luciferase Assay System (Promega, Madison, WI, United States) according to the manufacturer’s recommendations. The levels of luciferase activity were detected with a SpectraMax i3x microplate reader (Molecular Devices, CA, United States). Results were expressed per relative light units. The pGL4.13[luc2/SV40] vector containing SV40 early enhancer/promoter and pGL4.10[luc2] promoterless vector were included as positive and negative controls, respectively.

### Hepatocellular carcinoma gene expression profiling

The expression of 19 genes involved in hepatocellular carcinoma tumorigenesis were evaluated by qPCR. Total cellular RNA was extracted from control or sgtF1b Cosmopolitan and sgtF1b Basal transfected cells with TRIzol reagent (Invitrogen, Carlsbad, CA, United States). RNA samples were treated with RQ1 RNase-free DNase (Promega, Madison, WI, United States) at 37°C for 1 h, to remove DNA. Concentration and purity of the RNAs was determined by spectrometry. One microgram of RNA was reverse transcribed into cDNA with Random Hexamer Primers (Biodynamics, Buenos Aires, Argentina) using M-MLV reverse transcriptase (Promega, Madison, WI, United States). qPCR was performed using Luna Universal qPCR Master Mix (2x; New England Biolabs, Beverly, MA, United States). Primers used for the amplifications are detailed in Supplementary Table 2. The expression levels were normalized by five housekeeping genes: β-actin, GAPDH, LDHA, NONO and PPIH. Relative expression was calculated using the method of 2^−ΔΔCt^ (Riedel et al., 2014).

### Statistical analysis

All experiments were performed independently three times. Statistical significance was determined using a two-tailed Student t-test or one-way analysis of variance (ANOVA) followed by *post hoc* Tukey’s test. A value of *p* < 0.05 was considered to be statistically significant. Results were expressed as mean ± standard deviation. All analyses were performed using GraphPad Prism 8 software (GraphPad Software, San Diego, CA, United States).

## Results

### Molecular characterization of subgenotype F1b

In order to carry out an in-depth molecular characterization of the subgenotype F1b, full-length genome sequences derived from chronically infected patients were retrieved from GenBank (*n* = 99) and the maximum likelihood method was used to address the subgenotype F1b phylogeny.

**Figure 1:**
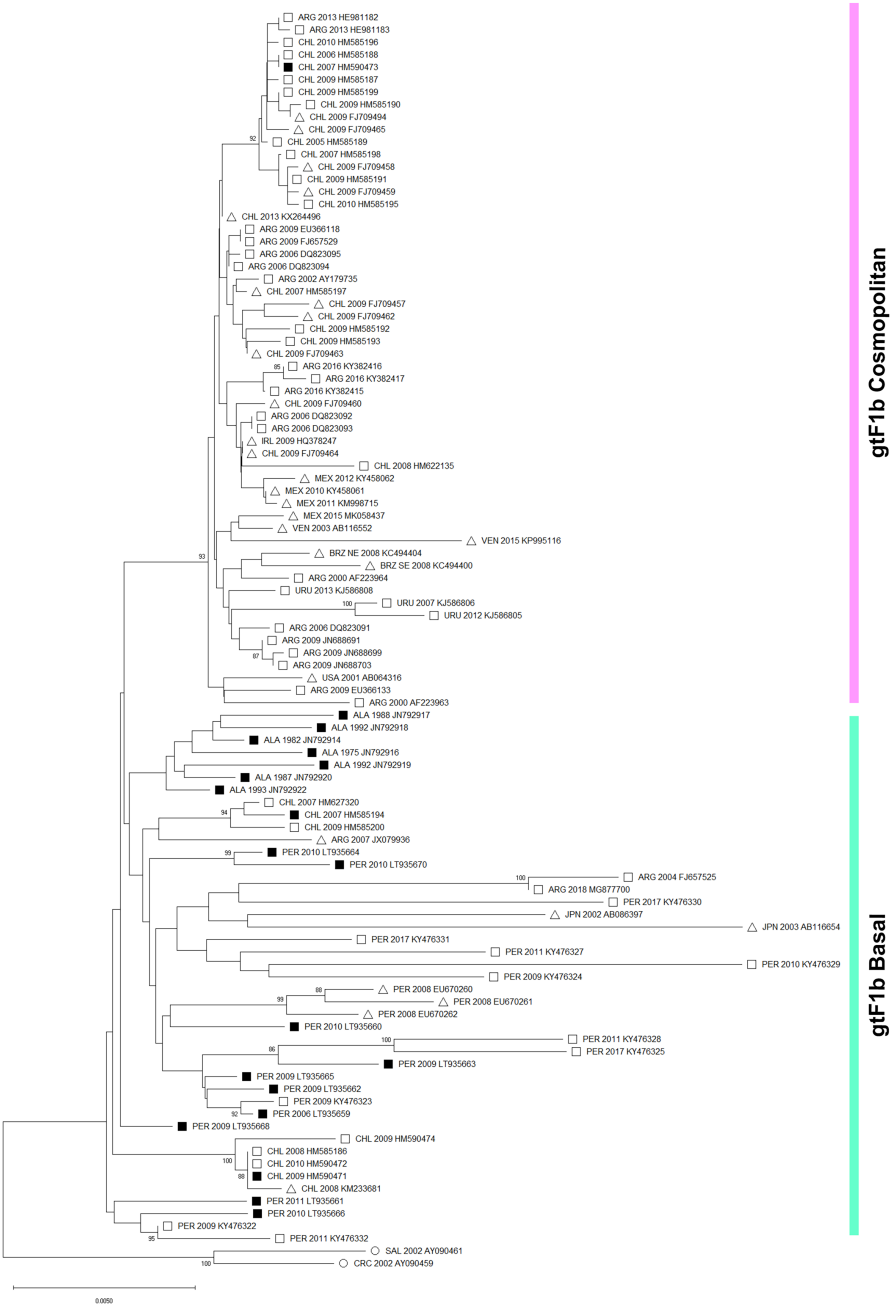
Phylogenetic analysis of subgenotype F1b sequences. A maximum likelihood tree was constructed on subgenotype F1b complete genome sequences derived from chronically infected patients (*n* = 99), using subgenotype F1a as an outgroup (*n* = 2, ○). The numbers at each node correspond to bootstrap values (greater than 90%) obtained with 1,000 replicates. The scale bar indicates the genetic distances. Sequences are name by the country of origin, followed by the year of the sample collection and the accession number. ALA, Alaska; ARG, Argentina; BRZ, Brazil; CHL, Chile; CRC, Costa Rica; IRL, Ireland; JPN, Japan; MEX. Mexico; PER, Peru; SAL, El Salvador; URU, Uruguay; USA, United states; VEN, Venezuela. ■ Nucleotide sequences associated with HCC cases. □ Nucleotide sequences non-associated with HCC cases. ∆ Data unknown.

The topology of the phylogenetic tree agreed with previous analyses of the whole genotype F and allowed to differentiate two groups within the subgenotype F1b (Figure 1). On the one hand, a strongly supported cluster designated as “Cosmopolitan,” which contains 56 sequences acquired largely from samples from Argentina and Chile and to a lesser extent from areas where the subgenotype F1b seems to be foreign, like Mexico, Uruguay, Venezuela, Brazil, Ireland, and the United States. On the other hand, lineages designated as “Basal” composed of 43 sequences obtained mainly from Peru, Chile, and Alaska. Furthermore, a different topology was observed between both groups. While the Basal lineages showed multiple internal monophyletic clusters from the same geographic location showing appreciable genetic distances among them, the Cosmopolitan cluster was homogeneous with highly similar sequences even when collected from distant geographic locations. The genetic distance between the clusters was 0.81. Of particular note, the identification of the subgenotype F1b nucleotide sequences associated with known HCC cases revealed that they all but one belonged to the gtF1b Basal group.

**Figure 2:**
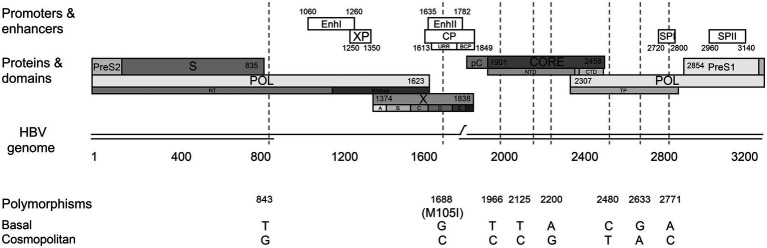
Differential nucleotide signature pattern between subgenotype F1b clusters. Schematic representation of HBV genome including enhancers I and II, promoters (white boxes) and proteins (gray boxes). Polymorphisms in nucleotide positions: 843, 1688, 1966, 2125, 2200, 2480, 2633, 2771 are shown.

Interestingly, the sequence alignment analysis of the subgenotype F1b full-length sequences allowed the identification of a differential signature pattern of eight nucleotides distributed throughout the genome, between the Cosmopolitan and Basal clusters (Figure 2). A sequence logo depicting the relative distribution of the 8 polymorphic nucleotides between the subgenotype F1b clusters is included in Supplementary Figure 1.

Four of these nucleotide polymorphisms were spotted in the viral polymerase (C/T 2480, G/A 2633, and A/C 2771 in the terminal protein domain and T/G 843 in the RT domain); three were detected in the core region (T/C 1966, T/C 2125, and A/G 2200) and one in the X gene (G/C 1688). The latter is the only non-synonymous polymorphism, leading to the M/I 105 change in the transactivation domain of the X protein (HBx). In addition, polymorphisms in positions 1,688 and 2,771 affected regulatory elements of HBV replication.

It must be taken into account that, even though the sequences belonging to both clusters possess these distinctive eight polymorphisms, most of the sequences also include other nucleotide variations characteristic of each particular sample.

### Biological characterization of subgenotype F1b clusters

In order to compare the biological characteristics of the subgenotype F1b clusters, samples closest phylogenetically to the consensus of the gtF1b Cosmopolitan and gtF1b Basal groups were selected for cloning and the replicative capacity, antigen expression levels and promoter activity of these clones was analyzed.

#### HBV replicative capacity differ between gtF1b Cosmopolitan and gtF1b Basal clones

To analyze the replication capacity of gtF1b Cosmopolitan and gtF1b Basal variants, three days’ post-transfection, cells and culture supernatants were harvested, and the levels of cccDNA, viral RNAs, intracellular HBV DNA, and secreted virion DNA were evaluated.

**Figure 3:**
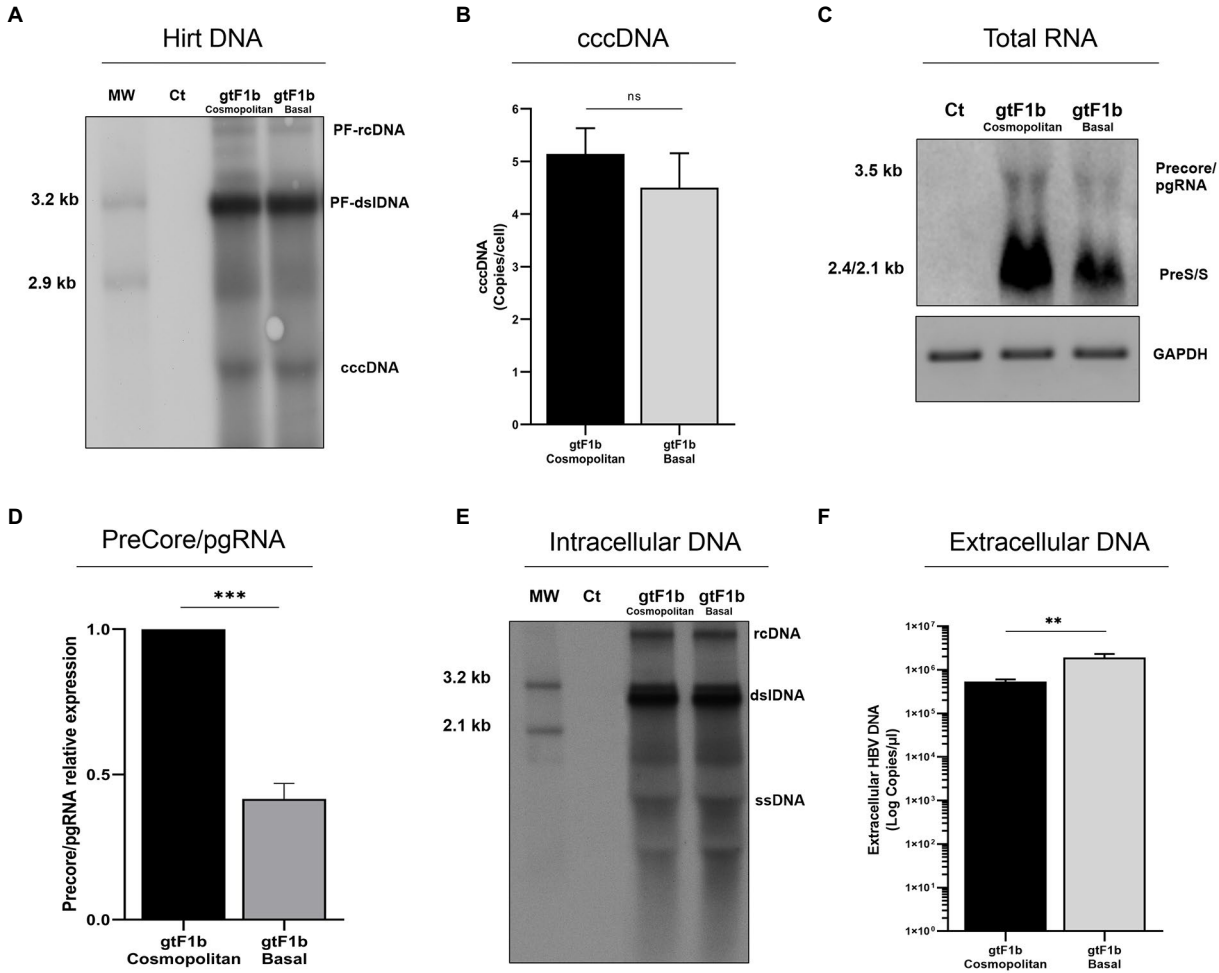
Analysis of HBV replicative capacity between gtF1b Cosmopolitan and gtF1b Basal variants. HuH-7 cells were transfected with linear full-length HBV genomes of gtF1b Cosmopolitan and gtF1b Basal variants and three days’ post-transfection cells and supernatants were harvested. **(A)** DNA was isolated after Hirt extraction and subjected to Southern blot analysis. **(B)** Total DNA was extracted, treated with T5 exonuclease to remove non-supercoiled dsDNA, and cccDNA levels were assessed by qPCR. Results were normalized to mitochondrial DNA. **(C)** Total RNA was extracted, and HBV RNA was detected by Northern blot. GAPDH RNA served as loading control. **(D)** Relative intensity of the Precore/pgRNA band was quantified using ImageJ software. **(E)** Cytoplasmic DNA was extracted HBV DNA replicative intermediates were assessed by Southern blot. **(F)** Extracellular HBV DNA was quantified by qPCR. PF-rcDNA: protein free HBV relaxed circular DNA; PF-dslDNA: protein free HBV double-stranded linear DNA. rcDNA: HBV relaxed circular DNA; dsLDNA: HBV double-stranded linear DNA; SS: HBV single-stranded DNA. Shown values represent the mean ± standard deviation of three independent experiments. ***p < 0.0001; **p < 0.005; ns: no statistical differences.

No significant differences were observed in cccDNA levels between gtF1b Cosmopolitan and gtF1b Basal clones by either Southern blot or qPCR (Figures 3A,B). Conversely, marked differences were detected in HBV RNA levels between both variants. The gtF1b Basal clone showed significantly lower levels of the 3.5 Kb Precore and pgRNA, and the 2.4/2.1 Kb preS/S RNAs, compared to the gtF1b Cosmopolitan clone (*p* < 0.0001; Figures 3C,D). Remarkably, the uneven RNA levels between both variants were not reflected in the intracellular HBV DNA levels, since no significant differences were observed between the gtF1b variants (Figure 3E), while extracellularly, the gtF1b Basal clone had significantly higher levels of HBV DNA in relation to the gtF1b Cosmopolitan clone (*p* < 0.0001; Figure 3F). Similar findings were also detected when these clones were transfected in HepG2 cells, although, as previously reported in several studies, viral load was substantially lower in HepG2 cells than in Huh7 cells (Supplementary Figure 2).

#### HBV protein expression and secretion varied between gtF1b Cosmopolitan and gtF1b Basal clones

To investigate the expression and secretion of HBV proteins between both variants, cells and culture supernatants were harvested from transfected cells and the extra and intracellular levels of HBsAg and HBeAg were examined.

**Figure 4:**
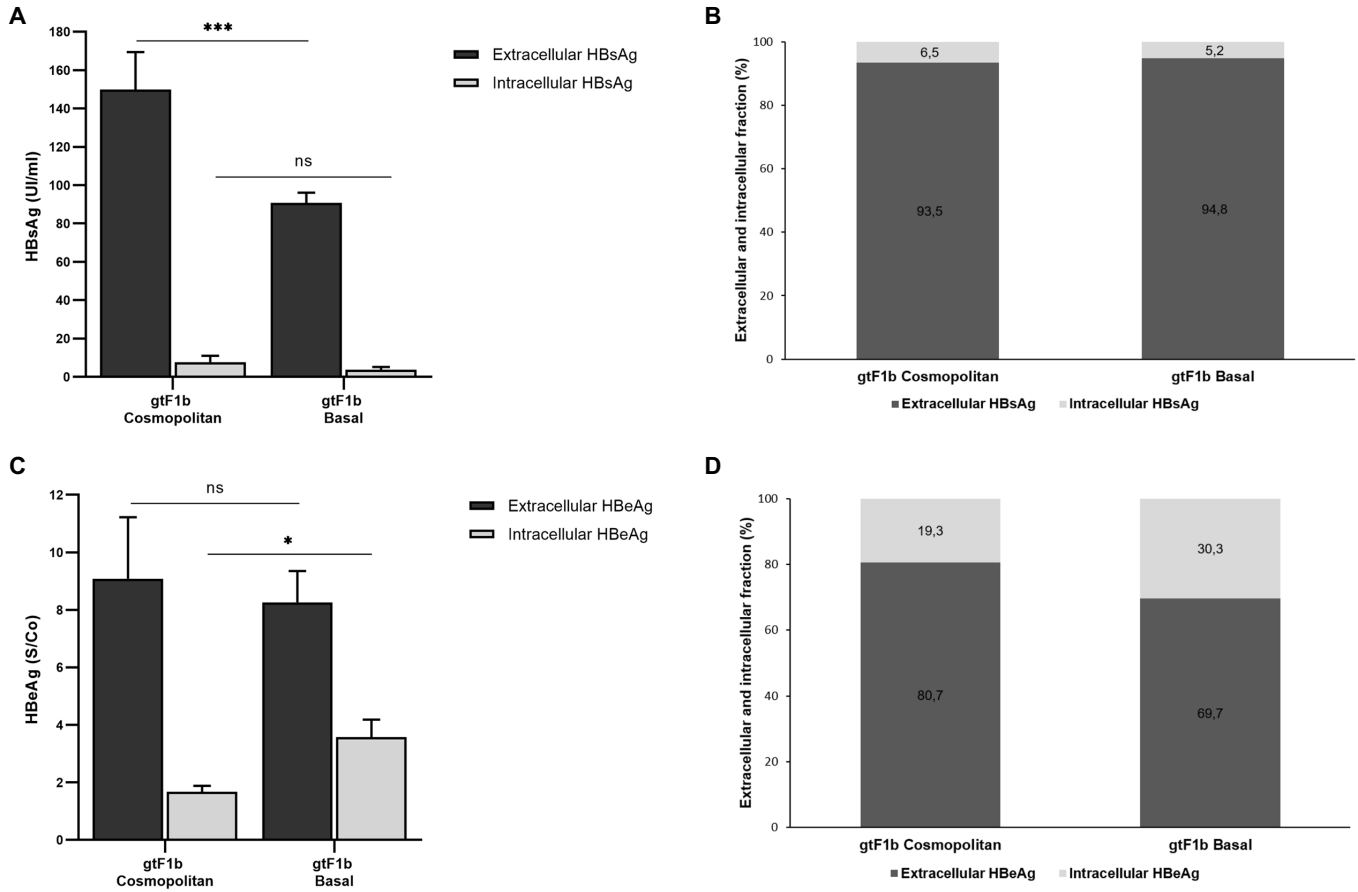
Analysis of intracellular and secreted HBsAg and HBeAg levels of gtF1b Cosmopolitan and gtF1b Basal variants. HuH-7 cells were transfected with linear full-length HBV genomes of gtF1b Cosmopolitan and gtF1b Basal variants. Three days’ post-transfection, cells and culture supernatants were harvested. Intracellular and extracellular levels of HBsAg and HBeAg were determined by ECLIA **(A,C)**. Extracellular/intracellular HBsAg and HBeAg ratio **(B,D)**. Values shown represent the mean ± standard deviation of three independent experiments. ***p < 0.0001; *p < 0.05; ns: no statistical differences.

At the intracellular level, no significant differences were observed in the levels of HBsAg between both clones, while at the extracellular level, the gtF1b Cosmopolitan clone secreted significantly higher levels of the antigen (*p* < 0.0001; Figure 4A). Regardless of the absolute amount of HBsAg, the ratio of intra/extracellular HBeAg showed that both clones secreted approximately 95% of the expressed antigen (Figure 4B). In a similar way, comparable levels of extra and intracellular HBsAg were observed in HepG2 cells (Supplementary Figures 3A,B).

On the other hand, regarding HBeAg, no statistical differences were observed in the extracellular levels between gtF1b Cosmopolitan and gtF1b Basal variants. On the contrary, the intracellular HBeAg levels were significantly higher in the gtF1b Basal clone compared to the gtF1b Cosmopolitan clone (*p* < 0.05, Figure 4C). Consequently, the intra/extracellular HBeAg ratio showed that gtF1b Basal retained intracellularly a greater proportion of the expressed antigen (Figure 4D). Similar patterns of HBeAg expression and secretion were also detected after transfection in HepG2 cells, though protein expression was considerably lower in HepG2 cells than in Huh7 cells (Supplementary Figures 3C,D).

To rule out the possibility of cross-reactivity of the Core protein and HBeAg, HuH-7 cells were also transfected with a full-length HBV genome harboring the G1896A Precore mutation that abrogates HBeAg expression. Intra and extracellular HBeAg was not detected in the Precore mutant (data not shown), indicating that there is no cross-reactivity between HBV Core protein and HBeAg in the ECLIA assay.

**Figure 5:**
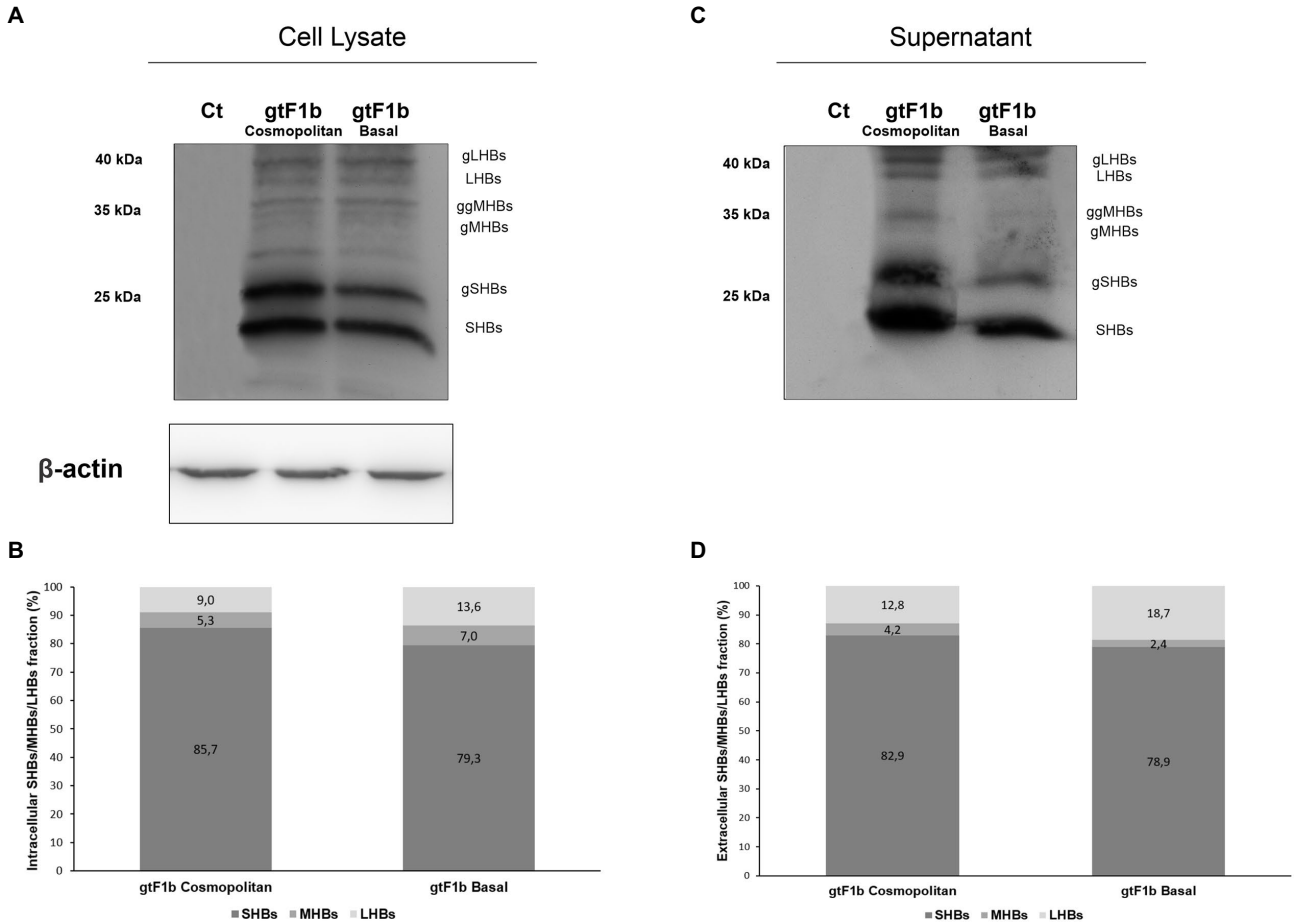
Relative composition of HBsAg proteins of gtF1b Cosmopolitan and gtF1b Basal variants. HuH-7 cells were transfected with linear full-length HBV genomes of gtF1b Cosmopolitan and gtF1b Basal variants. Three days’ post-transfection, cellular lysates **(A)** and the supernatants **(C)** were analyzed by Western Blot, using a SHBs-specific monoclonal (HB01) antibody. SHBs and LHBs occur in unglycosylated (LHBs and SHBs) and glycosylated (gLHBs and gSHBs) forms, while MHBs occurs in glycosylated (gMHBs) and double glycosylated (ggMHBs) forms. **(B)** Intracellular and **(D)** extracellular LHBs/MHBs/SHBs ratio was determined. Ct: cell transfected with pUC19 empty vector (control).

To further characterize the expression of HBsAg between variants, the relative abundance of the different surface proteins (LHBs, MHBs, and SHBs) was examined. For both clones, it was observed that SHBs (24 and 27 kDa) represented the major fraction of HBsAg, both intra and extracellularly (Figure 5). However, SHBs levels were higher for the gtF1b Cosmopolitan clone in relation to gtF1b Basal clone (Figures 5B,D). The relative amount of LHBs/MHBs compared to SHBs was higher, both intra and extracellularly, for the gtF1b Basal clone (20%) in comparison with the gtF1b Cosmopolitan clone (15%), while, both clones showed higher levels of LHBs (42 and 39 kDa) than MHBs (36 and 33 kDa), in the cell lysates and in the supernatant.

#### Differential HBV promoter activity between gtF1b Cosmopolitan and gtF1b Basal clones

To unravel the role of HBV promoters in the differential regulation of viral transcription between the gtF1b Cosmopolitan and gtF1b Basal variants, we characterized the activity of the SPI, SPII, pregenomic and Precore promoters. For this purpose, promoter sequences were inserted into the pGL4.10 vector and Luciferase reporter activity was determined.

**Figure 6:**
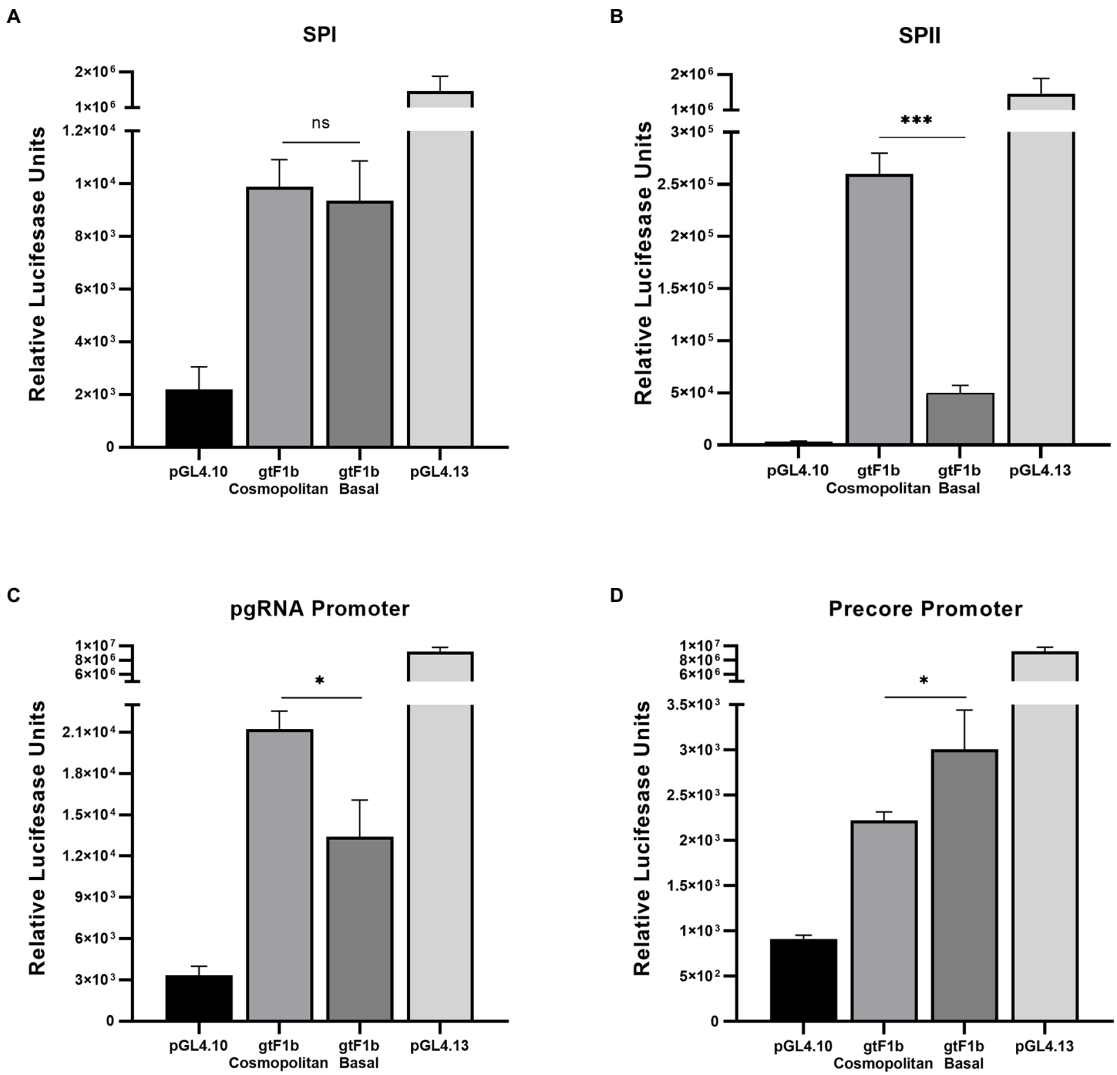
Analysis of HBV promoter activities of gtF1b Cosmopolitan and gtF1b Basal variants. HuH-7 cells were transfected with luciferase reporter constructs containing SPI **(A)**, SPII **(B)**, Core promoter with Precore TATA box-like mutated sequence **(C)**, and Core promoter with pgRNA TATA box-like mutated sequence **(D)** of gtF1b Cosmopolitan and gtF1b Basal variants. Three days’ post-transfection, cells were harvested, and luciferase activity was detected. Values shown represent the mean ± standard deviation of three independent experiments. The pGL4.13[luc2/SV40] and pGL4.10[luc2] vectors were included as positive and negative controls, respectively. *p < 0.05; ***p < 0.0001; ns: no statistical differences.

No significant differences were observed in the activity of the SPI promoter between gtF1b Cosmopolitan and gtF1b Basal variants (Figure 6A). However, around ten-fold stronger activity of the SPII promoter was detected in the gtF1b Cosmopolitan clone (*p* < 0.0001; Figure 6B).

It has been shown that the synthesis of the Precore and pregenomic RNAs in the Core promoter region is directed by two overlapping yet separate promoters, each consisting of its own transcriptional initiator and a TATA box-like sequence situated approximately 25 to 30 bp upstream of its sites of initiation (Yu and Mertz, 1996, 1997). To independently determine the activity of the Precore and pgRNA promoters, point mutations were introduced into the basal elements of the Precore or the pgRNA promoters in order to disrupt the activity of one of the promoters while not affecting the other. For both variants, a stronger activity of the pgRNA promoter was detected in comparison with the Precore promoter (Figures 6C,D), which indicates that the Core promoter activity is mostly driven to the synthesis of pgRNA. When compared the activity of the pgRNA promoter, gtF1b Cosmopolitan variants presented a stronger activity in relation to the gtF1b Basal variants (*p* < 0.05; Figure 6C). Whereas the analysis of the Precore promoter revealed the gtF1b Cosmopolitan clone had a weaker activity compared to the gtF1b Basal clone (*p* < 0.05; Figure 6D).

Taken together, these results showed that gtF1b Cosmopolitan and gtF1b Basal variants differ in their replication capacity, their protein expression and secretion levels as well as in the regulation of the activity of the viral promoters.

### Analysis of cellular pathways associated with carcinogenesis

In order to evaluate the modulation of signaling pathways closely associated with HCC tumorigenesis by the subgenotype F1b clusters, the mRNA expression levels of 19 genes implicated in the cellular pathways: Wnt/β-Catenin, TGFβ, NF-κB, EGF, VEFG, cell cycling, apoptosis and DNA damage response, was assessed by RT-qPCR.

**Figure 7:**
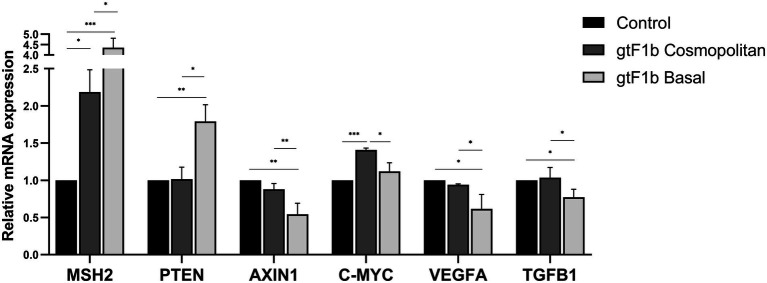
Expression analysis of HCC-related genes in gtF1b Cosmopolitan and gtF1b Basal variants. HuH-7 cells were transfected with pUC19 empty vector (control), or linear full-length HBV genomes of gtF1b Cosmopolitan and gtF1b Basal variants. Three days’ post-transfection, cells were harvested, total RNA was extracted, and mRNA levels of HCC-related genes were analyzed by RT-qPCR. Relative expression was calculated using the method of 2^−ΔΔCt^. Values shown represent the mean ± standard deviation of three independent experiments. *p < 0.05; **p < 0.005; ***p < 0.0001.

Of the 19 analyzed genes, 7 (36.8%) did not show significant differences in its expression levels among gtF1b Cosmopolitan and gtF1b Basal variants and control cells (Supplementary Figure 4A). Six genes (31.6%) were differentially expressed in both or at least one of the subgenotype F1b clusters, when compared to control cells (Supplementary Figure 4B). These include genes involved in apoptosis (BAX and FAS), cell cycling (CCND2) and EGF (EGF, AKT1 and PI3KCA) cellular pathways. Remarkably, a differential gene expression between gtF1b Cosmopolitan and gtF1b Basal variants was observed in the remaining six genes (31.6%; Figure 7). These comprise genes involved in DNA damage response (MSH2), Wnt/β-Catenin (AXIN1, C-MYC), TGFβ (TGFB1, AXIN1 and C-MYC) and VEGF (VEGFA and PTEN) cellular pathways. Similar findings were observed when the expression of these genes was analyzed in HepG2 cells, with the exception of C-MYC, where no significant differences were detected between variants (Supplementary Figure 5). Therefore, these results suggest that gtF1b Cosmopolitan and gtF1b Basal variants can modulate differentially the expression of genes associated with HCC progression.

## Discussion

Several studies conducted on the Alaskan native and Peruvian populations have described a close association between subgenotype F1b infection and the early appearance of HCC (Bertani et al., 2013; Ching et al., 2016; Gounder et al., 2016; Pineau et al., 2018; Hayashi et al., 2019). On the other hand, in Argentina, where subgenotype F1b is the most prevalent genotype, the HCC incidence rate is intermediate (less than 5.6 cases per 100,000 people/year), being HBV infection the etiological agent of only 5.4% of the HCCs (Piñero et al., 2018, 2020). This clinical-epidemiological discrepancy prompted us to perform a molecular characterization of the subgenotype F1b full-length genomes, from which two phylogenetically strongly supported clusters were identified. One of the clusters was designated as gtF1b Basal, and included sequences mostly from Alaska and Peru, while the other was called gtF1b Cosmopolitan and contained samples mainly from Argentina and Chile. Interestingly, the clusters were characterized by a differential signature pattern of eight nucleotides distributed throughout the genome.

Three of these polymorphisms were spotted in the amino terminal domain (NTD) of the Core gene (T/C 1966, T/C 2125, and A/G 2200). Mutations in this domain (T1938C and A2051C) have shown to markedly increase subgenotype F1b replication and up-regulate the expression of genes related to cell proliferation and carcinogenesis in Alaskan Native people with HCC (Hayashi et al., 2019). However, the frequency of these mutations in subgenotype F1b samples from Argentina was infrequent and mostly associated with HBeAg-negative chronic infections without HCC (Ledesma et al., 2015). Four other polymorphisms mapped into the Polymerase gene: three in the terminal protein domain (C/T 2480, G/A 2633, A/C 2771) and one in the RT domain (T/G 843). Although A/C 2771 polymorphism spotted in the SPI region, to which numerous transcription factors bind regulating the activity of the promoter (Kim et al., 2016), no differences between variants were observed neither in the SPI promoter activity, nor in the LHBs levels. Finally, G/C 1688 polymorphism spotted in the EnhII and the upper regulatory region (URR) of the Core promoter, which modulates the activity of the SPI, SPII, and X promoters and the transcription of the pg./Precore mRNAs, respectively (Quarleri, 2014). Several nuclear factors bind near the 1688 position (Li et al., 1995; Kim et al., 2016), consequently the polymorphism might alter its binding affinity. Simultaneously, this polymorphism implies a change in amino acid 105 of HBx. Remarkably, it was shown that a mutation at position 1,689, which also involved an amino acid change at 105 of HBx, was an independent predictive factor for HCC development (Kim et al., 2009).

Based on these findings, we carried out an exhaustive *in vitro* characterization of representative clones of each group in order to determine, on the one hand, the implication of these polymorphisms in the biology of the virus and, on the other hand, their impact on cellular pathways closely associated with HCC tumorigenesis.

In the present study, to mimic viral diversity, a mix of clones of each cluster was used. Most studies comparing HBV replicative capacity across genotypes have used only one clone per genotype/subgenotype; however, it has been shown that within the viral population of a sample there is a wide range of variants with significantly different biological characteristics (Parekh et al., 2003; Yang et al., 2004; Cui et al., 2010). In addition, it was observed that the sequence of one clone may not entirely match to the sequence in the biological sample (Hernández et al., 2014). On the other hand, it is not feasible to biologically characterized in-depth several clones in each sample. For these reasons, the biological characterization of a pool of clones from the same biological sample, as previously described (Qin et al., 2011), makes it possible to evaluate the replication phenotype and the antigen expression capacity of the majority of the viral population.

The gF1b Basal clone presented significantly lower levels of pgRNA/Precore mRNAs in comparison with the gF1b Cosmopolitan clone. This could be associated with the lower activity observed in the pgRNA promoter in the gF1b Basal clone, although, this no support the difference observed in the Precore promoter activity. Despite the difference in pgRNA levels, the amount of intracellular HBV DNA was similar in both clones, and even higher levels of extracellular DNA were detected in the gtF1b Basal clone. These findings suggest regulations at different stages of the viral replication that go beyond pgRNA transcription. This may include from factors affecting the packaging and/or reverse transcription of pgRNA to the variations in the sequence of the regulatory elements and/or trans-regulation between different viral proteins that might contribute to the differential replication phenotype observed in both clones.

The analysis of antigen expression and secretion also revealed substantial differences between both variants. The gtF1b Basal clone expressed significantly lower levels of HBsAg than the gtF1b Cosmopolitan clone. In this regard, the study of the promoter activities revealed a weaker SPII activity in the gtF1b Basal clone, as well as a lower level of the preS/S mRNAs, which might be responsible for the reduced HBsAg production. Furthermore, the study of HBeAg expression revealed that, while no differences were observed in the extracellular levels, the gtF1b Basal clone showed significantly higher levels of intracellular HBeAg. This could also be associated with the higher activity of the Precore promoter in the gtF1b Basal clone.

Interestingly, even though similar patterns of secreted virus and HBeAg expression were observed in Huh-7 and HepG2 cells lines, no significant differences between both clones were detected in HepG2 cells. It must also be taken into account that the viral load, as well as the protein expression levels were substantially lower in HepG2 cells than in Huh7 cells. These results suggest that cellular factors characteristic to each cell line might differentially modulate viral replication and protein expression. In according with this, previous studies have reported that Huh-7 cells are known to facilitate HBx-independent HBV replication (Yaginuma et al., 1987) whereas, and in HepG2 cells, replication of the HBV is HBx-dependent (Zoulim et al., 1994; Bouchard et al., 2003). Therefore, it is important to perform the biological characterization of genotypes/subgenotypes in more than one cell line to validate the results.

Finally, since the deregulation of cellular pathways has been widely described as one of the main mechanisms that contribute to HCC development (Farzaneh et al., 2021) we performed a characterization of cellular pathways closely associated with HCC tumorigenesis.

On the one hand, the gtF1b Basal clone showed a significant increase in the mRNA levels of MSH2 and PTEN in relation to the gtF1b Cosmopolitan clone. In this context, it has been reported that MSH2 overexpression induced genome instability phenotypes which might be important for promoting cancer progression, including HCC (Lin et al., 2016; Chakraborty et al., 2018). On the other hand, the expression of four other genes (TGFB1, VEGFA, c-Myc and AXIN1) was down-regulated in the sgtF1b Basal clone. TGFB1 is a pleiotropic cytokine with multifaceted roles. It has been demonstrated that HBV can up-regulate Smad7 expression and prevent TGB1 signaling, which inhibits apoptosis and promotes tumorigenesis in liver cells (Liu et al., 2015). VEGFA plays a crucial role in the control of angiogenesis, including tumor development and progression. The down-regulation of VEGFA could be a consequence of the increased levels of PTEN, which has been reported as a VEGFA repressor (Roh et al., 2003). c-Myc, a highly pleiotropic transcription factor, is a gene driver of malignant transformation in primary stages of HCC. Likewise, c-Myc binds to the VEGFA promoter, activating its transcription. Therefore, its negative regulation could also contribute to the decreased levels of VEGFA observed in the gtF1b Basal clone. Finally, AXIN1 is a multidomain scaffold protein. It was demonstrated that HBV-related HCCs present a lower expression of AXIN1 in relation adjacent non-tumor tissues (Li et al., 2013), suggesting that low expression levels of AXIN1 might contribute to the oncogenic potential of the gtF1b Basal cluster.

Overall, these results suggest that beyond genotypes and subgenotypes, even variants with few nucleotide differences, show dissimilar biological properties that might alter the progression of the infection. Although it cannot be overlooked that host factors, as well as mutations that naturally occur in the course of infection might influence the outcome of chronic infection (Liu et al., 2009; Trinks et al., 2017; Zhang et al., 2019), the fact that multiple HBV genotypes co-circulate in Alaska, where genetic and dietary factors are homogeneous among the native population, but primordially the gtF1b Basal cluster is closely associated with HCC (Livingston et al., 2007; McMahon et al., 2021), supports that the polymorphisms identified in this cluster might contribute to a worse progression of infection. It must also be considered that the paucity of clinical data, as well as the underlying demographics and risk factors for HCC in Latin America represent a limitation for the high-certainty analysis. Therefore, it would be advisable to carry out more studies that characterized the clinical-epidemiological setting of subgenotype F1b infections in the region to further address the role of these polymorphisms in the progression of the infection.

One limitation of the study is that the main findings were obtained on hepatoma cell lines (Huh-7 and HepG2). Although these cell lines have been widely used to characterize virus-cell interactions, and to study cellular pathways (Liu et al., 2015; Datta et al., 2018; Khatun et al., 2018; Hayashi et al., 2019), many intracellular pathways might be altered in relation to primary hepatocytes, and therefore extrapolation of the findings obtained should be taken with caution. Future studies will utilize primary human hepatocytes a more biologically relevant system to study the differential modulation of cellular pathways associated with HCC by the subgenotype F1b clusters.

## Conclusions

This study identified two subgenotype F1b clusters with distinct molecular and biological properties. Furthermore, a differential regulation in the expression of genes associated with HCC tumorigenesis was identified, which might account for the dissimilar progression of infection of subgenotype F1b clusters. Hence, this work contributes to unravel the controversy regarding the outcome of subgenotype F1b HBV chronic infections.

## Data availability statement

The datasets presented in this study can be found in online repositories. The names of the repository/repositories and accession number(s) can be found at: https://www.ncbi.nlm.nih.gov/genbank/, OK106257; https://www.ncbi.nlm.nih.gov/genbank/, OL907123.

## Author contributions

MME performed the experiments, analyzed the data, and drafted the manuscript. LMo, MS, BB, LT, and LMa contributed to the conduction of experiments. MME, RHC, and DMF participated in the conception, drafting, and/or editing of the manuscript. All authors have read and agreed to the published version of the manuscript.

## Funding

This work was supported by grants from Agencia Nacional de Promoción Científica y Tecnológica (ANPCyT) [PICT 2019-01456], and Universidad de Buenos Aires: UBACyT [20020170100206BA 2018-2021], PIDAE [2019-3473].

## Conflict of interest

The authors declare that the research was conducted in the absence of any commercial or financial relationships that could be construed as a potential conflict of interest.

## Publisher’s note

All claims expressed in this article are solely those of the authors and do not necessarily represent those of their affiliated organizations, or those of the publisher, the editors and the reviewers. Any product that may be evaluated in this article, or claim that may be made by its manufacturer, is not guaranteed or endorsed by the publisher.
