# Supplementary material for: Molecular and biological characterization of hepatitis B virus subgenotype F1b clusters: Unraveling its role in hepatocarcinogenesis
Source: Front Microbiol. 2022 Jul 27;13:946703. doi: 10.3389/fmicb.2022.946703 (PMC9363773; doi:10.3389/fmicb.2022.946703)
Supplement: Supplementary file 1 [file Data_Sheet_1.DOCX]

**SUPPLEMENTARY MATERIAL**

**Supplementary Table 1. Primers for the construction of Luciferase reporter vectors.**

| **Name** | **Sequence 5'-3'** | **Annotation** |
| --- | --- | --- |
| CP_Fw | CTTCGCGG**GAGCTC**CTTTGTTTA | Bold: *SacI* restriction site |
| CP_Rv | AAGGCACA**GCTAGC**AGGCTTGAA | Bold: *NheI* restriction site |
| SPI_Fw | GTATTCCTT**GAGCTC**ATAAGGKGG | Bold: *SacI* restriction site |
| SPI _Rv | CCCATGC**GCTAGC**TCTTGTTC | Bold: *NheI* restriction site |
| SPII_Fw | GGATTCTTTC**GAGCTC**AYCAGYTGG | Bold: *SacI* restriction site |
| SPII _Rv | CCTYCCK**GCTAGC**CGATTGGTG | Bold: *NheI* restriction site |
| PcMut_Fw | GAGATTAGGT**CCC**AGGTCTTTGTA | Bold: preCore TATAbox-like mutation |
| PcMut_Rv | TACAAAGACCT**GGG**ACCTAATCTC | Bold: preCore TATAbox-like mutation |
| PgMut_Fw | AGGAGGCTGTAGGCAT**TG**A**C**TGGT | Bold: pgRNA TATAbox-like mutation |
| PgMut_Rv | ACCA**G**T**CA**ATGCCTACAGCCTCCT | Bold: pgRNA TATAbox-like mutation |

**Supplementary Table 2. Primers for relative quantification of genes involved in hepatocellular carcinoma tumorigenesis.**

| **Gene** | **Sequence 5'-3'** |
| --- | --- |
| **Bax** | Fw: ATGGGCTGGACATTGGACTTC |
|  | Rv: GATGGTGAGTGAGGCGGTGAG |
| **Fas** | Fw: AATGCCCAAGTGACTGACATC |
|  | Rv: GGGCTTTGTCTGTGTACTCCT |
| **MHS2** | Fw: CTACGATGGATTTGGGTTAGC |
|  | Rv: TGCGATTCTCCAATATACTGA |
| **CCND2** | Fw: GAGGAACAGAAGTGCGAAGAA |
|  | Rv: CATGGCAAACTTAAAGTCGGT |
| **CDKN2A** | Fw: AACCTCGGGAAACTTAGAT |
|  | Rv: ATGGACATTTACGGTAGTGGG |
| **PTEN** | Fw: CAGAAAGACTTGAAGGCGTAT |
|  | Rv: ATTTGGCGGTGTCATAA |
| **c-MYC** | Fw: GCTGCTTAGACGCTGGATTT |
|  | Rv: CACCGAGTCGTAGTCGAGGT |
| **AKT1** | Fw: CACAGCCCTGAAGTACTCTT |
|  | Rv: CCCGAAGTCTGTGATCTTAAT |
| **NFKB1** | Fw: CACAGCCCTGAAGTACTCTT |
|  | Rv: CCCGAAGTCTGTGATCTTAAT |
| **TGFB1** | Fw: ACAAGCCCAGAGAGGTTAAGG |
|  | Rv: TGCTAGGATTACAGGCGTGAG |
| **SOCS3** | Fw: CAAGGACGGAGACTTCGATT |
|  | Rv: GGAGCCAGCGTGGATCTG |
| **AXIN1** | Fw: CCCCAAAGTCTGTAGTGAC |
|  | Rv: ATTGACATAATAGGGGTTGAC |
| **SMAD4** | Fw: GTCTTTGATTTGCGTCAGT |
|  | Rv: TGATGCTCTGTCTTGGGTAAT |
| **VEGFA** | Fw: CTACTGCCATCCAATCGAGAC |
|  | Rv: GGTTTGATCCGCATAAT |
| **PIK3CA** | Fw: CACGCAGGACTGAGTAACAGA |
|  | Rv: ACAGAAGCAATTTGGGTAGA |
| **PDGFRA** | Fw: CTATGTGCCAGACCCAGATGT |
|  | Rv: GTTACAGGAGTCTCGGGATCA |
| **GADD45B** | Fw: GCAACATGACGCTGGAAGAG |
|  | Rv: GGATGAGCGTGAAGTGGATT |
| **EGF** | Fw: TTCCTCTTAGCCCAGTATCCT |
|  | Rv: AAGACCTTCTGGCACATCTAC |
| **PTK2** | Fw: TCAAGAATAACGGAAGGGAGA |
|  | Rv: GGTTGGCTCACTATTGCTTTC |
| **LDHA** | Fw: CCAATATGGCAACTCTAAAGG |
|  | Rv: ACCAAATTAAGACGGCTTTC |
| **NONO** | Fw: AATCTTCCTCCCGACATCACT |
|  | Rv: ATGACTACAGCCCTCTCTACC |
| **PPIH** | Fw: GATGTCAGTATTGGCGGTCAGG |
|  | Rv: AAGAACTGACAGCCATTTGTA |
| **GAPDH** | Fw: CTCTGACTTCAACAGCGACAC |
|  | Rv: AGCCAAATTCGTTGTCATAC |
| **ACTB** | Fw: CAAAGACCTGTACGCCAACAC |
|  | Rv: GTCATAGTCCGCCTAGAA |


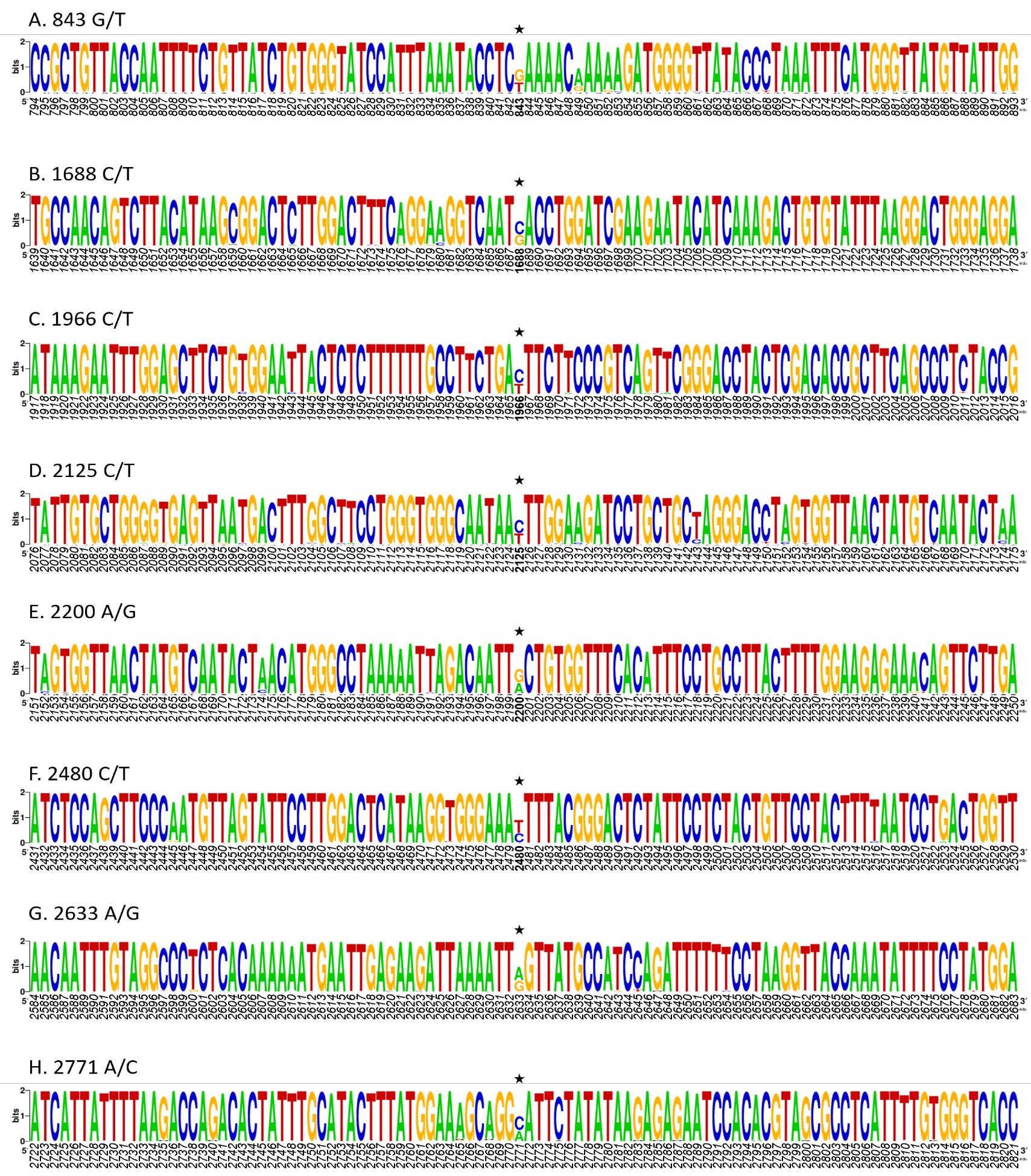


**Supplementary Figure 1.** Sequence logo depicting the relative distribution of the 8 polymorphic nucleotides (star) across the two subgenotype F1b clusters. Each polymorphic position +/- 50 nucleotides are shown. A. 843 G/T polymorphism, B. 1688 C/T polymorphism, C. 1966 C/T polymorphism, D. 2125 C/T polymorphism, E. 2200 A/G polymorphism, F. 2480 C/T polymorphism, G. 2633 A/G polymorphism, and H. 2771 A/C polymorphism.

**

**

**Supplementary Figure 2.** Analysis of HBV DNA levels of gtF1b Cosmopolitan and gtF1b Basal variants. HepG2 cells were transfected with linear full-length HBV genomes of gtF1b Cosmopolitan and gtF1b Basal variants. Three days’ post-transfection culture supernatants were harvested and HBV extracellular DNA levels were determined by qPCR. Shown values represent the mean ± standard deviation of three independent experiments. ns: no statistical differences.





**Supplementary Figure 3.** Analysis of intracellular and secreted HBsAg and HBeAg levels of gtF1b Cosmopolitan and gtF1b Basal variants. HepG2 cells were transfected with linear full-length HBV genomes of gtF1b Cosmopolitan and gtF1b Basal variants. Three days’ post-transfection, cells and culture supernatants were harvested. Intracellular and extracellular levels of HBsAg and HBeAg were determined by ECLIA (A, C). Extracellular/intracellular HBsAg and HBeAg ratio (B, D). Values shown represent the mean ± standard deviation of three independent experiments. **: p < 0.005; ns: no statistical differences.


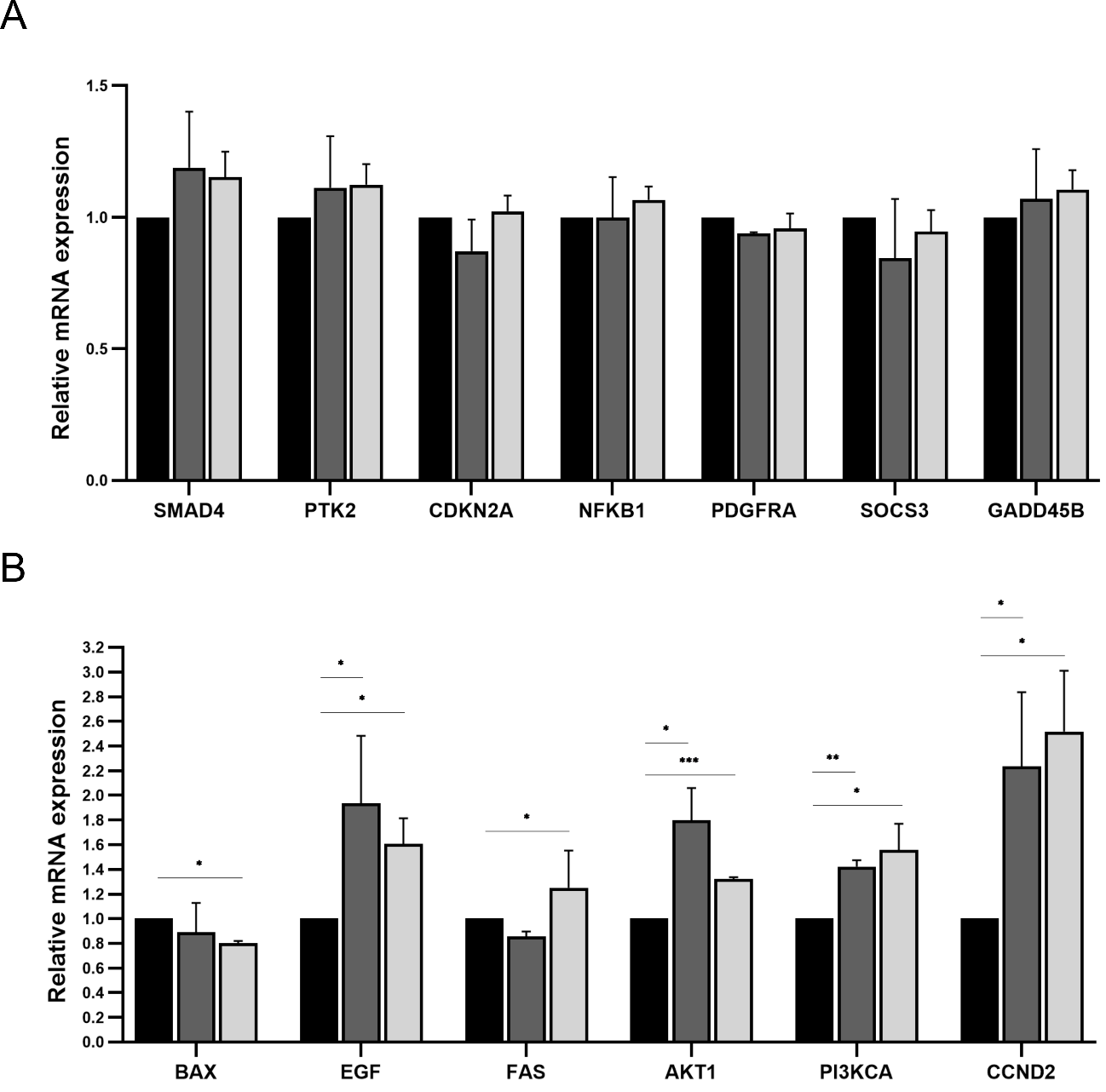


**Supplementary Figure 4.** Expression analysis of HCC-related genes in gtF1b Cosmopolitan and gtF1b Basal variants. HuH-7 cells were transfected with pUC19 empty vector (control, ) or linear full-length HBV genomes of gtF1b Cosmopolitan () and gtF1b Basal () variants. Three days’ post-transfection, cells were harvested, total RNA was extracted, and mRNA levels of HCC-related genes were analyzed by RT-qPCR. Relative expression was calculated using the method of 2^−ΔΔCt^. (A) No statistical difference in relation to control cells. (B) Statistical difference in relation to control cells. Values shown represent the mean ± standard deviation of three independent experiments. *: p < 0.05; **: p < 0.005; ***: p < 0.0001.

**
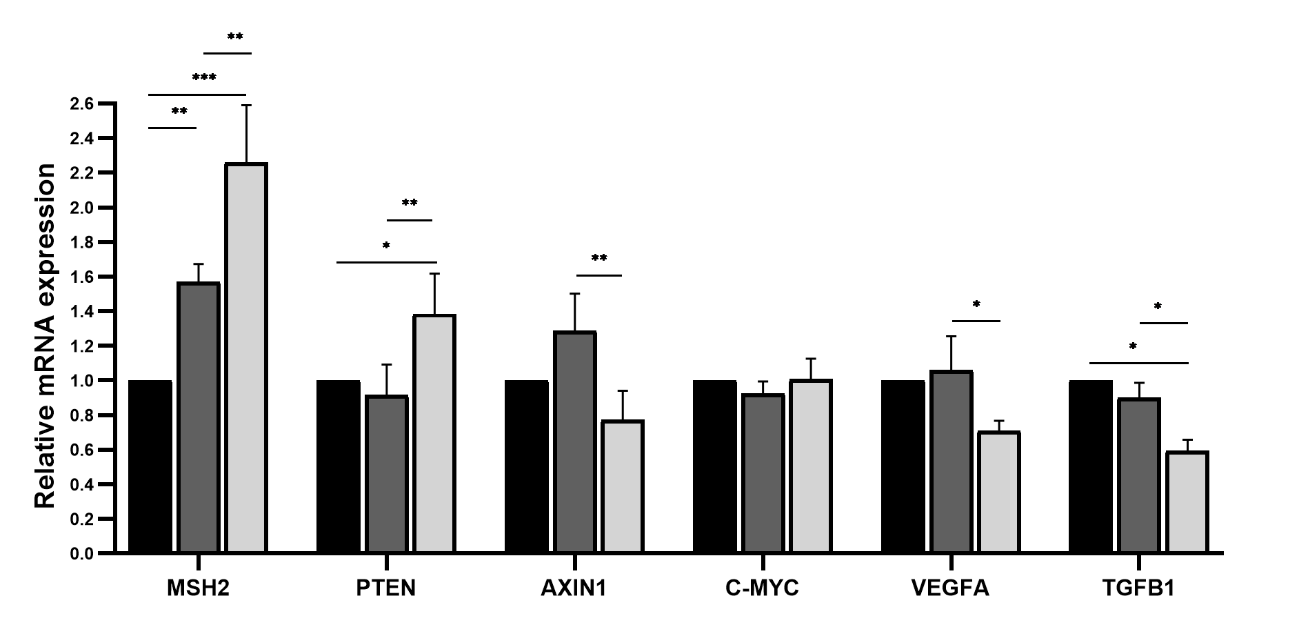
**

**Supplementary Figure 5.** Expression analysis of HCC-related genes in gtF1b Cosmopolitan and gtF1b Basal variants. HepG2 cells were transfected with pUC19 empty vector (control, ) or linear full-length HBV genomes of gtF1b Cosmopolitan () and gtF1b Basal () variants. Three days’ post-transfection, cells were harvested, total RNA was extracted, and mRNA levels of HCC-related genes were analyzed by RT-qPCR. Relative expression was calculated using the method of 2^−ΔΔCt^. Values shown represent the mean ± standard deviation of three independent experiments. *: p < 0.05; **: p < 0.005; ***: p < 0.0001.
