# Supplementary figures and images for: Molecular and biological characterization of hepatitis B virus subgenotype F1b clusters: Unraveling its role in hepatocarcinogenesis
Source: Front Microbiol. 2022 Jul 27;13:946703. doi: 10.3389/fmicb.2022.946703 (PMC9363773; doi:10.3389/fmicb.2022.946703)

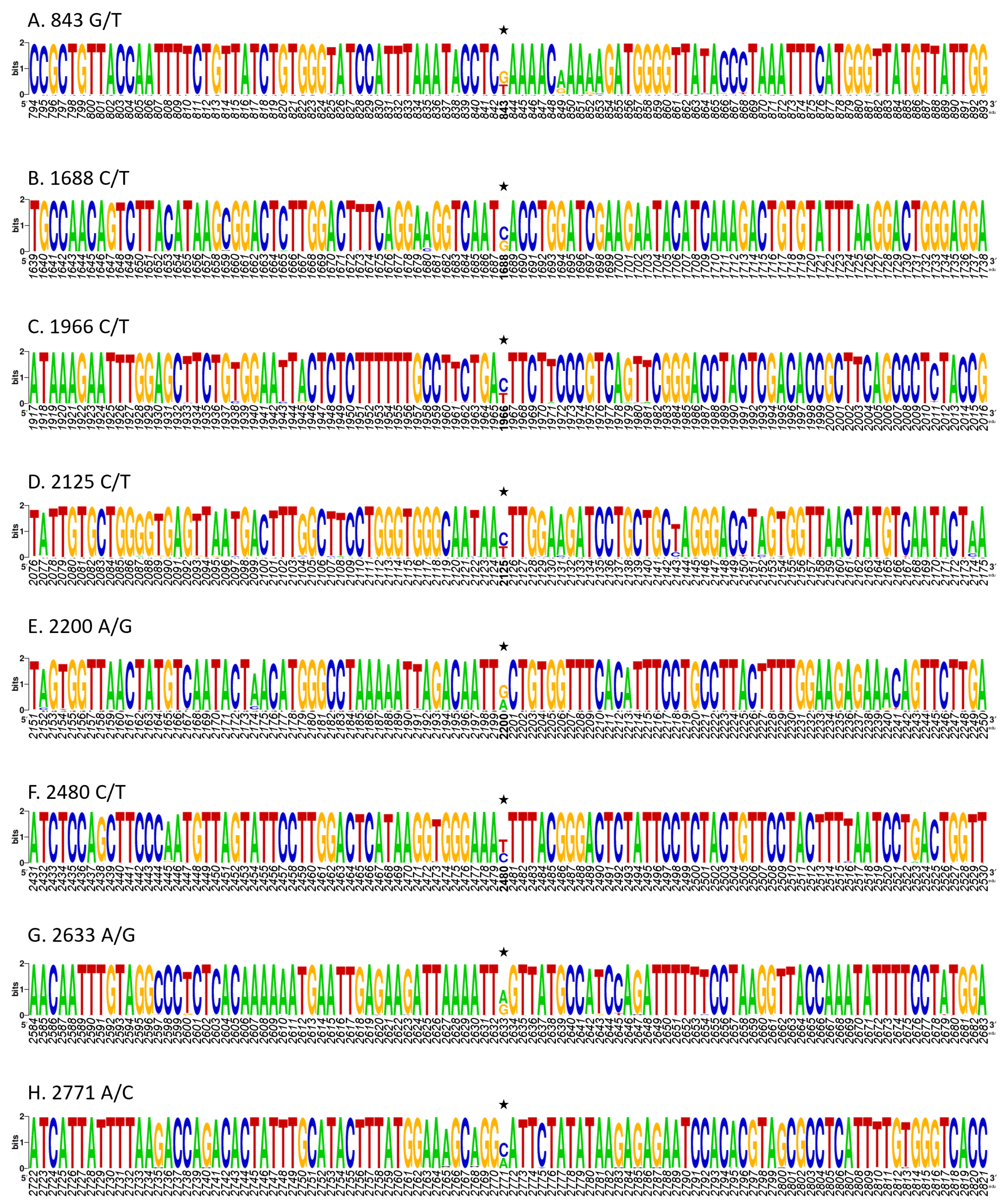

Supplement: Supplementary file 2 [file Image_1.TIF]

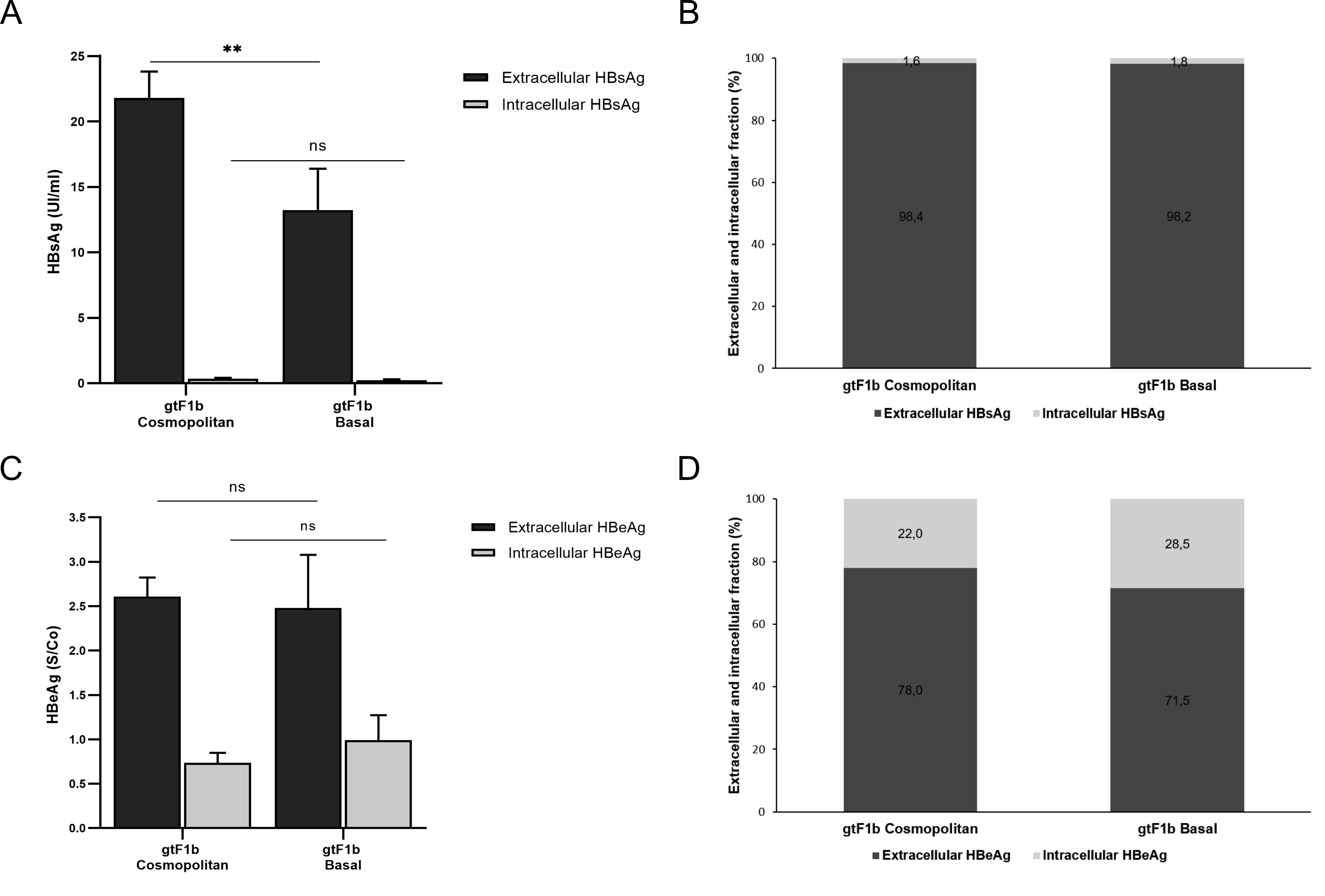

Supplement: Supplementary file 3 [file Image_2.TIF]

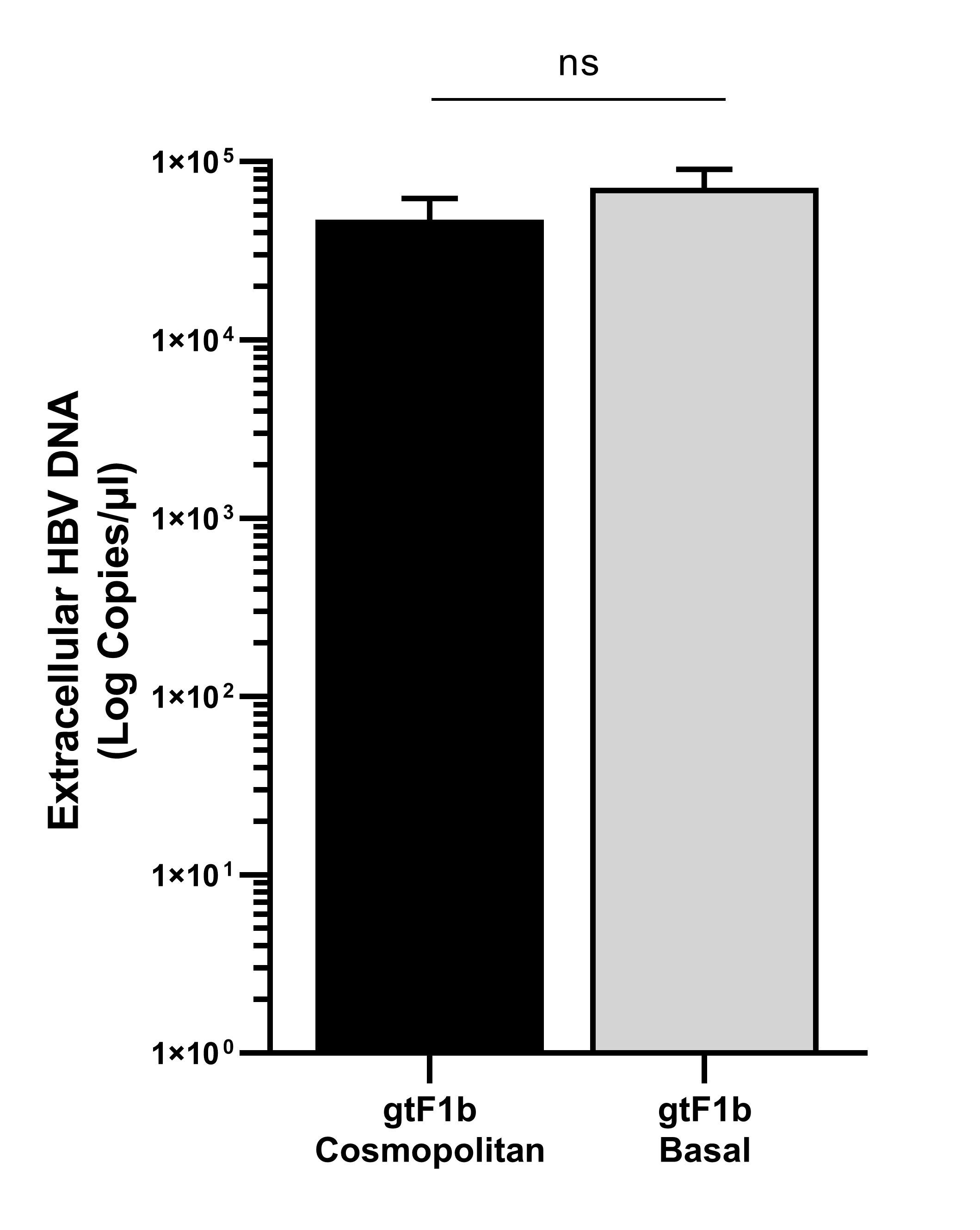

Supplement: Supplementary file 4 [file Image_3.TIF]

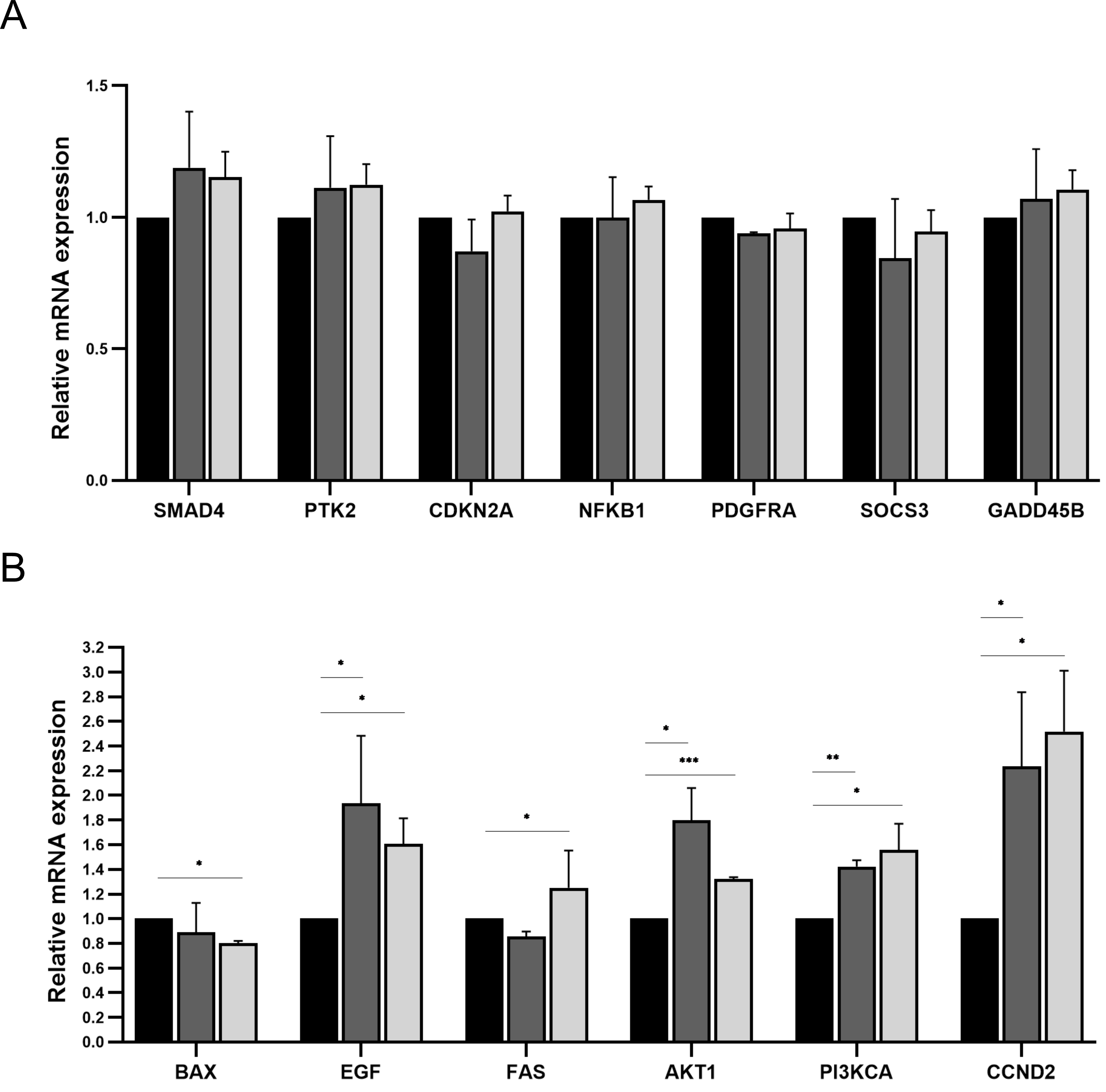

Supplement: Supplementary file 5 [file Image_4.TIF]

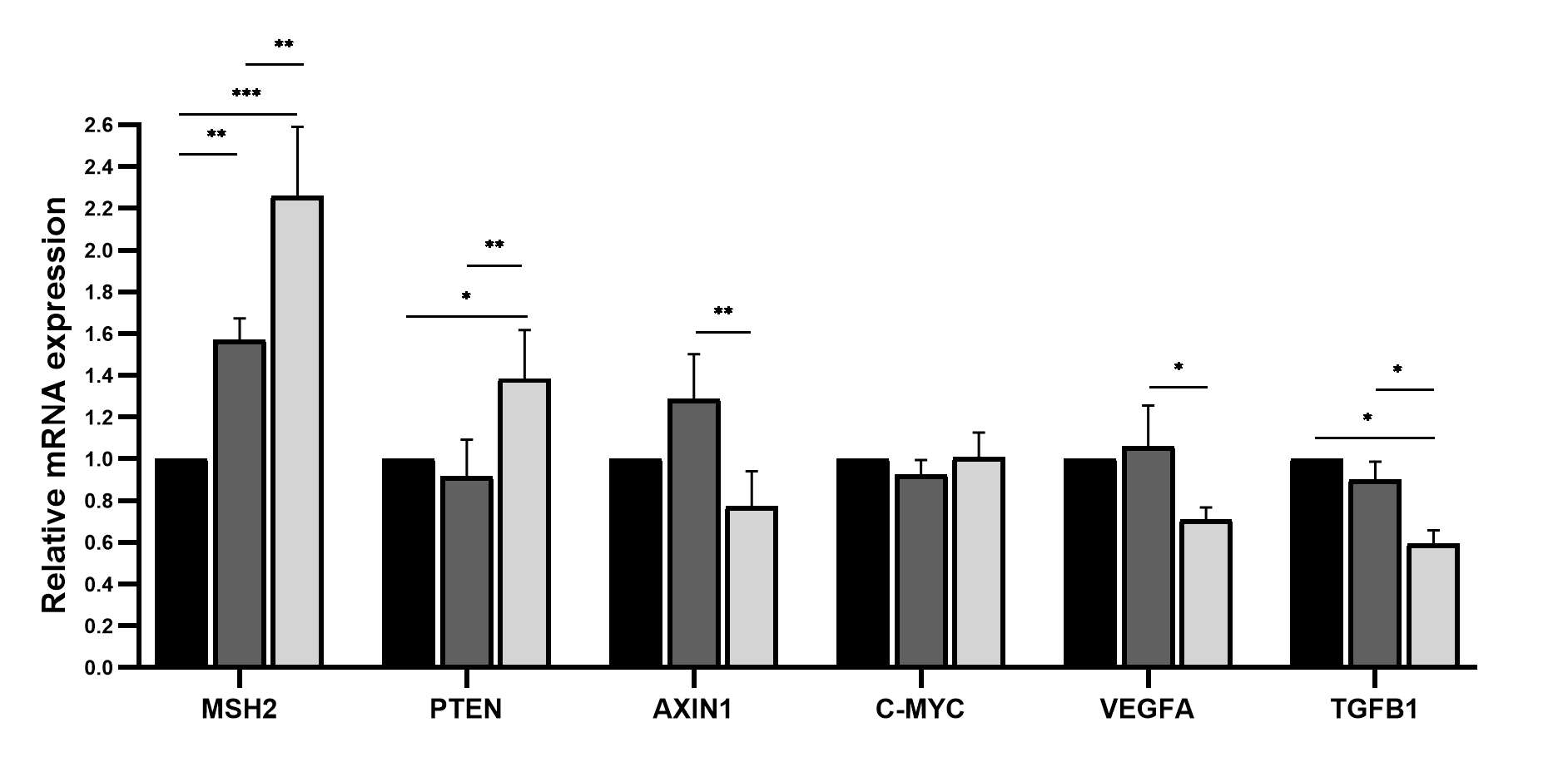

Supplement: Supplementary file 6 [file Image_5.TIF]
